# Naive Forecast for COVID-19 in Utah Based on the South Korea and Italy Models-the Fluctuation between Two Extremes

**DOI:** 10.3390/ijerph17082750

**Published:** 2020-04-16

**Authors:** Fares Qeadan, Trenton Honda, Lisa H. Gren, Jennifer Dailey-Provost, L. Scott Benson, James A. VanDerslice, Christina A. Porucznik, A. Blake Waters, Steven Lacey, Kimberley Shoaf

**Affiliations:** 1 Division of Public Health, Department of Family and Preventive Medicine, University of Utah, Salt Lake City, UT 84108, USA; fares.qeadan@utah.edu (F.Q.); Lisa.gren@hsc.utah.edu (L.H.G.); Jennifer.Dailey-Provost@utah.edu (J.D.-P.); scott.benson@hsc.utah.edu (L.S.B.); jim.vanderslice@utah.edu (J.A.V.); christy.porucznik@utah.edu (C.A.P.); Blake.Waters@Utah.edu (A.B.W.); steven.lacey@utah.edu (S.L.); Kimberley.shoaf@utah.edu (K.S.); 2 Division of Physician Assistant Studies, Department of Family and Preventive Medicine, University of Utah, Salt Lake City, UT 84108, USA

**Keywords:** COVID-19, pandemic, predictive modeling

## Abstract

Differences in jurisdictional public health actions have played a significant role in the relative success of local communities in combating and containing the COVID-19 pandemic. We forecast the possible COVID-19 outbreak in one US state (Utah) by applying empirical data from South Korea and Italy, two countries that implemented disparate public health actions. Forecasts were created by aligning the start of the pandemic in Utah with that in South Korea and Italy, getting a short-run forecast based on actual daily rates of spread, and long-run forecast by employing a log-logistic model with four parameters. Applying the South Korea model, the epidemic peak in Utah is 169 cases/day, with epidemic resolution by the end of May. Applying the Italy model, new cases are forecast to exceed 200/day by mid-April, with the potential for 250 new cases a day at the epidemic peak, with the epidemic continuing through the end of August. We identify a 3-month variation in the likely length of the pandemic, a 1.5-fold difference in the number of daily infections at outbreak peak, and a 3-fold difference in the expected cumulative cases when applying the experience of two developed countries in handling this virus to the Utah context.

## 1. Introduction

The key to preparation and appropriate response lies in a reasoned and well-devised plan that anticipates future needs. At times, this anticipated need is understood, such as flu vaccines prior to flu season. However, on occasion, there arises a new threat that is dynamic and less predictable based upon prior experience. The recent pandemic of SARS-CoV 2 is one such example. For the past 30 years, we have seen the introduction of viruses to the western hemisphere and the rest of the world where the population is naive to said viruses. These include Dengue, West Nile, SARS, Chikungunya, p (2009) H1N1, and now SARS-CoV2. Each virus arrived into non-immune populations and the successful or unsuccessful control of these viruses was dependent on the public health measures taken.

The present COVID-19 pandemic began in the city of Wuhan in Hubei province, China at the end of 2019 [1], with the first cases reported to the World Health Organization (WHO) China office on 31 December 2019 [2]. In response to the initial epidemic and in anticipation of potential international spread, the WHO issued guidance on assessing national capacities to guide countries on their response effort [3]. By 13 January 2020, a mere two weeks after the initial report to the WHO, the first international case of COVID-19 was reported in Thailand. By 30 January 2020, 98 cases were reported in 18 countries, including a number involving community transmission [4]. By April, there were over 1,000,000 cases across the world.

The present COVID-19 pandemic is a new and rapidly evolving outbreak. However, the differences in public health actions across affected countries are evident, as are the subsequent increases, and in some cases, decreases, in new cases. For example, South Korea responded early with proactive and aggressive containment measures. This included the development of rapid testing capabilities within two weeks of the first reported case, and within a month, closure of public spaces and schools, the implementation of extensive drive-through testing, and universal airport screening. Importantly, South Korea also implemented robust case tracking and contact tracing, and a protocol to isolate infected individuals. This rapid response appears to have dramatically limited the spread and severity of COVID-19 in South Korea, where the current case fatality rate is <1% [5].

Italy was surprised by the sudden presentation of cases within the northern regions of the country, which led to a more reactive and less proactive response. The unexpected and sudden increase in cases led to delays in testing, case tracking, contact tracing, and isolation of infected individuals, ultimately allowing community spread to take hold and devastate the population in the affected area. The first quarantines were not established until three weeks after the first case was observed. Possibly, as a result of the dramatic community spread overwhelming the local healthcare infrastructure, the case fatality rate in Italy is over eight times higher than that observed in South Korea (7.9%) [5].

One important approach to manage the anticipated future needs of a new and novel infectious disease, such as COVID-19, is the mathematical modeling of the epidemic curve to predict the future burden of disease. The best mathematical models are not mere calculations done in the absence of present data, rather they are the result of well-reasoned experience based on past events that are expressed in a way that provides some measure of predictability of future events. As the United States appears to be 1–2 weeks behind Europe in the arrival of the pandemic, by applying the experiences, assumptions, and response types from both South Korea and Italy with what has been observed locally in the state of Utah, one can set boundaries and expectations and begin to understand the impact of preparations, and what responses are appropriate to mitigate the devastation caused by COVID-19. Below, we model the predicted progress of COVID-19 in Utah by applying observed patterns of the spread of COVID-19 in South Korea and Italy based on publicly available data, and use these, with the utility of statistical modeling, to provide a range of estimates of the timing and magnitude of the spread of COV-19 in this region.

## 2. Materials and Methods

### 2.1. Theoretical Model

**Figure 1:**
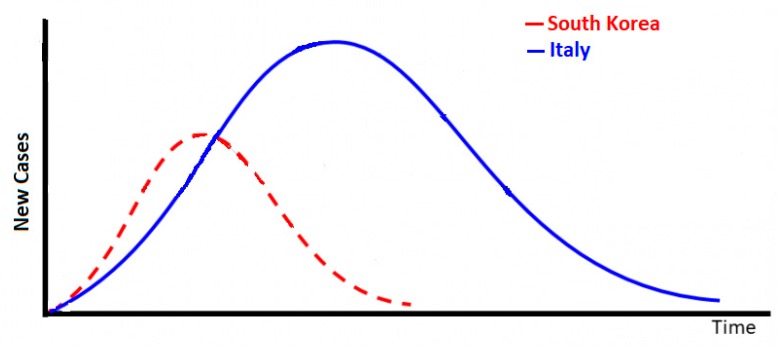
Hypothetical spread of new cases of the COVID-19 virus over time in a pandemic demonstrating two extremes, one with strong containment measures (South Korea) and another with weak containment measures (Italy).

Our general methodology to forecast COVID-19 in Utah—and by extension, other geographic jurisdictions—is naïve in the sense that it is based on existing daily rates of new cases from two countries representing extremes with respect to controlling the pandemic, namely, South Korea and Italy. We assume that the number of new cases in both countries rises rapidly, peaks, and then declines according to the epidemic curve. However, the rates at which each country reaches the peak (determining the acceleration phase of the pandemic), and declines after its peak (determining the deceleration phase of the pandemic) characterizes the extent of control over the pandemic. From empirical data, there is strong indication that the spread of COVID-19 in South Korea and Italy should follow a hypothetical epidemic curve that demonstrates these two extremes (see Figure 1).

### 2.2. Data Sources

Data on the daily new cases of COVID-19 for Utah, South Korea, and Italy were obtained from several online sources including: (1) The Utah Department of Health (UDOH) online reporting system for the COVID-19 surveillance [6]. This reporting system presents immediate reporting for positive cases from all major private and public laboratories in Utah, while noting that some cases could experience up to a 72-h lag before being reported. (2) The Worldometers website [2], which shows real-time global statistics at https://www.worldometers.info/coronavirus/, relies on many sources but mainly governmental entities such as state departments of health and it has been cited as a source in over 10,000 published books, and in more than 6000 professional journal articles. (3) Our World in Data website [7] by Max Roser at https://ourworldindata.org/ is a scientific online publication that focuses on large global problems; it is both open-access and open-source.

### 2.3. Model Forecast Procedures

We followed five steps to build the forecast model: (1) determine how many days Utah is behind South Korea and Italy in reporting new cases. For example, many experts report that the U.S. is between one and two weeks behind Italy. In our approach, to align the start date of the pandemic in Utah relative to South Korea and Italy, we overlapped the first seven consecutive days with non-zero cases in Utah (starting on 11 March) with that in South Korea (16 February) and Italy (20 February) (see Table 1). Thus, Utah lags behind South Korea and Italy by 24 and 20 days, respectively. (2) The second step of the forecast is to predict the number of new cases in Utah, for a number of days into the future that is equal to the number of days representing the lag between Utah and South Korea or Italy based on each country’s actual daily spread rates. Spread rates are calculated as the relative change in the number of new cases between two consecutive days. A list of the needed daily spread rates required for predicting new cases in Utah for the next 24 (South Korea) and 20 (Italy) days, is shown in Table 1. Since the actual daily spread rate is the relative change in new cases between two consecutive days by construction, it could be viewed in the sense of the Markovian property [8] as the number of cases tomorrow are assumed to depend only on today’s number of cases (i.e., P(X_t_|X_t−1_,…,X_1_)=P(X_t_|X_t−1_)). (3) The third step of the forecast is to calculate the cumulative number of cases for Utah, while allowing non-consecutive zero entries at the beginning of the spread. We first start with reported data (i.e., the cumulative daily sum of new cases from March 4th through April 4th in Utah, as reported by Utah Department of Health), and then end with the predicted data based on step-II (i.e., the continued cumulative daily sum of new cases from 5 April through 28 April based on the South Korea model and 24 April based on the Italy model. (4) The fourth step of the forecast is to fit a log-logistic model with four-parameters [9,10,11] for the cumulative number of cases, generated in step-3. This helps calculate the expected time for the peak of the pandemic (i.e., the incidence turning point) and expected time for the full carrying capacity of the outbreak (i.e., the total number of cumulative cases encountered throughout the pandemic). Log-logistic growth models are commonly used to model outbreaks, as they are able to capture the slow initial growth of the pandemic, followed by a period of rapid growth, and a period of slowing growth. These models have a characteristic sigmoidal (s-shaped) shape and mathematically have the functional form

(1) f(t)=c+(d−c)/(1+exp(b×(log(t)−log(e)))

where *f(t)* is the infected population size, *t* is time, *c* is the initial infected population size, and *d* is the carrying capacity, or final infected population size [12]. In the model, *e* indicates the inflection point of the logistic curve (i.e., the incidence turning point) and *b* is a slope parameter, which acts as a scaling factor [13]. (5) The fifth step of the forecast is to produce a smooth extrapolation of the growth curve model using time points beyond those in step-III. For example, the extrapolation to predict new cases in Utah will start on 25 April or 29 April, assuming the Italy or South Korea models, respectively. The drc library in the R statistical package [14] with drm() function was used to implement the four parameter log-logistic model (LL.4). For implementation purposes, one could use the R syntax:

*model = drm(y ~ x, fct = LL.4())* (2)

where *x* is time and *y* is cumulative cases.

## 3. Results

### 3.1. Model Using South Korea’s Experience

**Figure 2:**
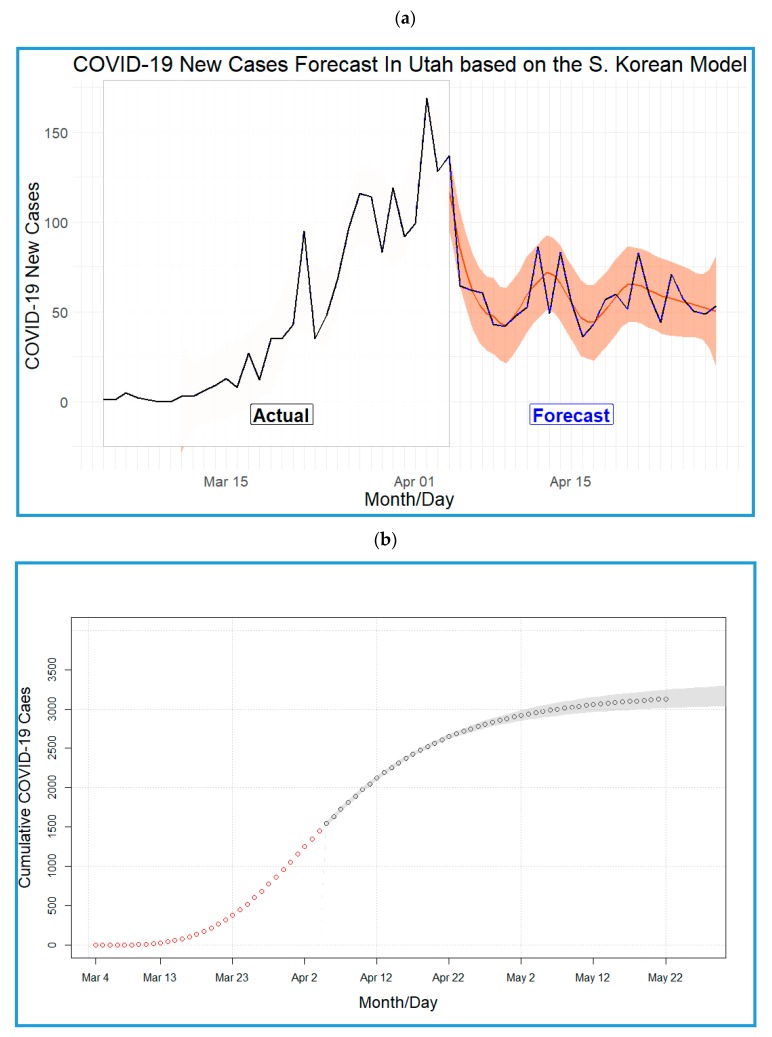
(**a**) Number of COVID-19 new cases in Utah (actual and forecasted) based on the South Korean model. (**b**) Predicted cumulative COVID-19 cases in Utah based on the South Korea model (red) and log-logistic (gray) models.

The largest number of new cases per day in Utah is 169, as shown in Figure 2a, which is an observation from the actual data rather than the predicted data, indicating that Utah has passed the peak according to this model. From the forecast, we expect fewer than 169 new cases per day in the following weeks. The four-parameter log-logistic model of the pandemic in Utah following the South Korea model is shown in Figure 2b (i.e., step III) utilizing the parameters’ estimates b = −3.83, c = −1.99, d = 3244.52, and e = 33.85. The COVID-19 pandemic in Utah is expected to end by the end of May according to the South Korea model with a total number of 3245 cases.

### 3.2. Model Using Italy’s Experience

**Figure 3:**
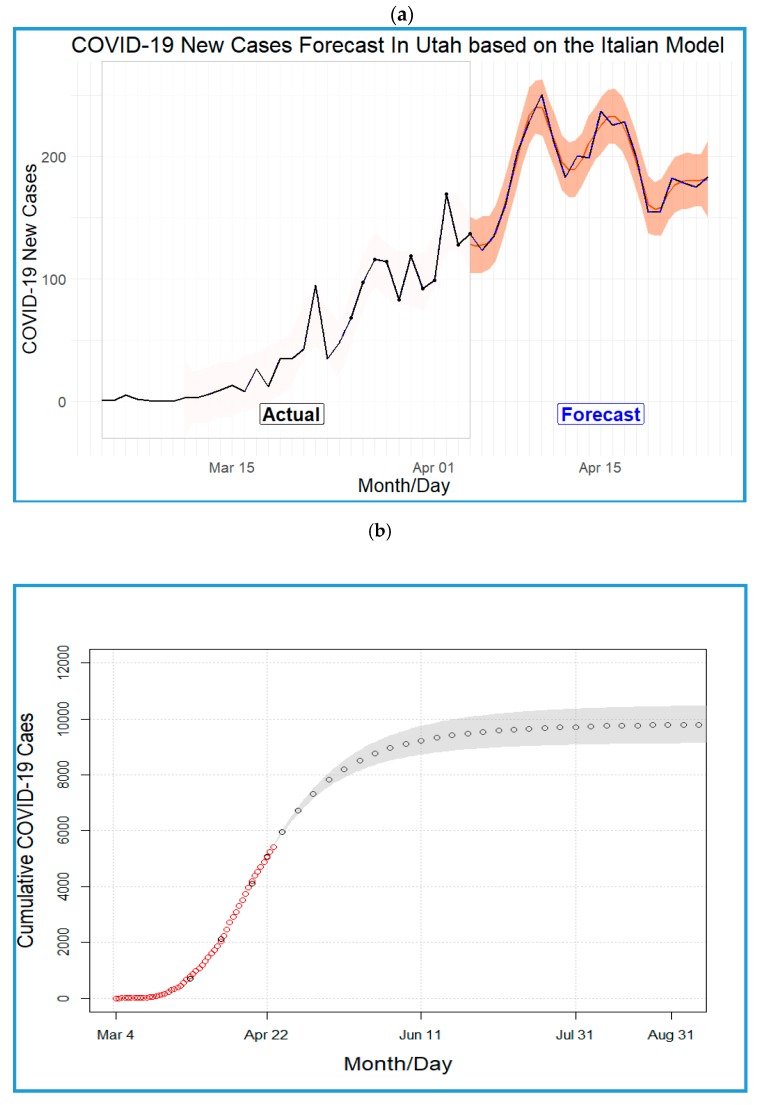

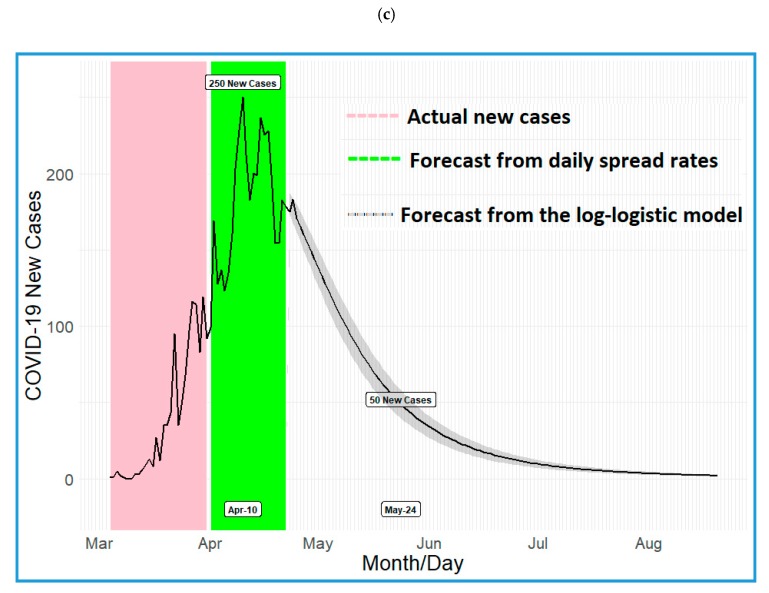
(**a**) Number of COVID-19 new cases in Utah (actual and forecasted) based on the Italy model. (**b**) The cumulative COVID-19 cases in Utah based on the Italy and log-logistic models. (**c**) Number of COVID-19 new cases in Utah (actual and forecasted) based on the Italy and log-logistic models.

More extreme forecasts for Utah are shown in Figure 3a–c, where the outbreak has not yet peaked but new cases are predicted to exceed 200/day by the second week of April (Figure 3a). Further, if the rate of spread stays consistent with what has been observed in Italy, the log-logistic forecast indicates that by 10 April, Utah might experience 250 new cases a day (Figure 3c). The carrying capacity or final infected population size in Utah is expected to be around 9857 (parameter estimates of the log-logistic model are b = −3.80, c = 6.71, d = 9856.90, and e = 49.18). Based on this forecast, the COVID-19 pandemic in Utah is expected to end by the end of August (Figure 3b,c).

### 3.3. Comparing Actions across South Korea, Italy and Utah

South Korea had their first confirmed case of COVID-19 on 22 January, and on 3 February, a diagnostic test was approved for use in the country (Table 2). Their response to the outbreak was centered on testing with more than 66,000 tests being administered by the end of February [15]. Intensive testing identified 3150 cases by 29 February and was accompanied by isolation and quarantine of cases/contacts as well as general implementation of social distancing measures in the country that included phone apps that tracked the location of individuals who tested positive.

Widespread drive-through testing was available by 29 February and screening of all individuals coming into the country, including citizens, was implemented 16 March.

Italy had their first confirmed case of COVID-19 on 31 January and immediately suspended flights to China and declared a national emergency (Table 2). The initial cases in January and February were imported cases and no further actions were taken until two clusters of cases were identified in Northern Italy starting on 20 February. On 23 February, the small towns involved in these clusters were placed under quarantine and carnival celebrations and football (soccer) matches were canceled. Over the ensuing time, additional quarantine and social distancing measures have been put into place. However, while Italy has conducted close to 300,000 tests for SARS Co-V2, there has not been an intensive coordinated effort of testing for case tracking and contact tracing. On 26 February, Italy announced that they would only be testing individuals with symptoms, which was a fact substantiated by the fact that 25% of their tests were positive, higher than all other reporting countries except Spain (with 50% of tests positive) [15].

Actions in Utah to address the Coronavirus pandemic were informed, in part, by observations of global experiences and outcomes. With the benefit of anticipating the arrival of the infection, decision makers in Utah implemented orders such as the soft closure of public schools for a two-week period beginning 14 March. Prior to the expiration of that two-week order, on 23 March, the school closure was extended to 1 May. Over the course of several weeks following the first confirmed Coronavirus cases (associated with travel and community acquisition on 6 March and 14 March respectively) government leaders at both the state and municipal levels implemented a variety of patchwork policies including closure of restaurants and bars for dining in (take-out and delivery continue to be available) and restriction of public gatherings. To date, no state-wide stay-at-home order has been implemented, though some county and city governments have issued orders and/or proclamations.

## 4. Discussion

In the current study, we identify a three-month range in the likely length of the pandemic in Utah, a 1.5-fold difference in the peak number of daily infections and a 3-fold difference in the expected cumulative number of cases when applying empirical data from South Korea and Italy to the Utah context. These two models in many respects represent a best-case and a worst-case scenario for the COVID-19 pandemic experience in the State of Utah assuming containment measures stay in place. The question is, why are these two models so different and how does Utah (or any other US state) act to ensure that the local experience is one of the best cases possible? There are multiple potential explanations for the variation in case number and disease spread between South Korea and Italy, including differences in demographic characteristics (e.g., Italy has the second-oldest population in the world) [45] or even different viral strains [46]. However, one likely factor is the different approaches to outbreak management between the two countries. Unlike demographic characteristics or rapidly evolving viral strains, the public health response is something that Utah and other localities anticipating the virus can control.

The containment strategy used in South Korea was based on widespread testing (case tracking), isolation, contact tracing, as well as a combination of social distancing, travel restrictions, and enforced quarantine. This approach was also successfully deployed in Hong Kong and Singapore in order to rapidly contain outbreaks [47]. The realization that many of the early cases in South Korea were associated with a single organization likely facilitated an initially focused intense response which was then broadened throughout the country. This containment strategy works best in relatively small populations with good public health and healthcare infrastructure, where administering and analyzing tests and contact tracing are feasible. Italy’s response was largely a “lockdown” strategy, which included mandatory quarantine, shelter-in-place orders or recommendations, bans on public gatherings, and closure of businesses. The success of this strategy depends on maintaining the isolation of individuals – whether self-imposed or enforced – and can be highly variable across geographic jurisdictions. Further, this approach typically results in additional waves of cases as restrictions are lifted, as has been seen in China, Hong Kong, Singapore, and Taiwan. These locations have reintroduced restrictions, in order to address the upswing in cases subsequent to relaxing the initial restrictions [47]. Thus, the lockdown strategy is most beneficial in terms of slowing the epidemic but, alone, does not lead to containment. In Utah, adequate containment of viral spread, seen to be beneficial elsewhere through mandatory shelter-in-place orders, has not been realized, likely due to the inconsistency of such orders. Further, Utah was slow to gain access to and implement wide-spread testing, possibly creating an exacerbation of community spread, only beginning large-scale testing on 19 March.

Another major difference between South Korea and Italy is their respective levels of geographic isolation. The geographic isolation of South Korea limits the generalizability of this country’s experience to the United States. Entry into South Korea via a land border is extremely limited, as the only land border is the Demilitarized Zone with North Korea. South Korea is well connected to the rest of the world via air travel, but the number of airports with an international service is relatively small. As it is a peninsula, there is a long oceanic border, but South Korea does not have close neighbors by sea. This geographic isolation may be an important, non-modifiable factor explaining some of the differences in the containment and control of the viral outbreak between South Korea and other countries. Italy is also a peninsula, but it is in the heart of Europe with many connections to other countries by land, air, and sea. The geographic situation for Italy is more similar to most U.S. states than the South Korean context. As a land-locked state which also serves as a transportation hub in the Intermountain West, Utah’s geography and unlimited restriction on interstate transportation add to challenges associated with the movement of individuals. Additionally, the stark inconsistency of prevention measures across municipalities and jurisdictions within the state further complicate the success rates of various policies. For example, Salt Lake City, the state’s capitol and largest city, issued a stay-in-place proclamation. The proclamation, however, applies only to those living within the city’s political boundaries, and jurisdiction of the rule does not extend to those who travel to the city for work or other reasons.

The potential impact of these two models on Utah are substantially different. With the South Korea model, Utah will have a pandemic peak around the beginning of April, with a likely maximum of 169 new cases per day and a cumulative total of around 3245 cases. With the Italy model, Utah would peak the second week of April with a maximum of around 250 new cases per day and a cumulative total of upwards of 9857 cases over the six months of the pandemic. In reviewing the difference in strategies, testing figures heavily into the equation. South Korea sought out and tested those who were potentially exposed and used the public health tools of quarantine and isolation. Italy depended primarily on the public health tools of extreme social distancing and testing of symptomatic cases only. This would suggest that for Utah to have an experience closer to South Korea, extensive and coordinated testing would be in order. The testing should focus primarily on contacts of cases with quarantine of exposed individuals and isolation of those testing positive.

While the start of the COVID-19 pandemic was in China, we decided to use other countries as a reference for the two extremes in our modeling for several reasons. First, the stability of the initial numbers from China were questionable given that it was not clear then that a new pandemic has been emerging. Second, we believe that COVID-19 behaves in a self-similar fashion geospatially in its spread trajectory or growth with the understanding that the size of the exposed population and the timing of containment measures at the beginning of the spread could modify the peak numbers and cumulative numbers of the pandemic. In fact, a recent study has shown that the number of infected people with time in the most impacted and unprepared countries in urban settings, indeed exhibit an asymptotic power law behavior confirming the self-similar property or fractal time growth of the COVID-19 pandemic [48]. Thus, there is no loss of generality in choosing Italy over China as one of the extremes in our modeling.

### Strengths and Limitations

The uniqueness of our approach is that it uses available daily rates of spread within South Korea and Italy to predict the total number of new cases in Utah for the lagging days in the pandemic between Utah and those two countries. The novel aspect of this approach is the use of case studies of outbreaks of the same organism in the recent past to provide rapid estimates of the potential impact in areas lagging in the development of the pandemic. In this way, one can expand the training set used in the fit of the cumulative number of cases, plotted as a function of time, as the only input necessary for the forecast in the log-logistic growth model. While such an approach may provide fairly accurate estimates for the times nearer to the date of extrapolation, it is less robust for further extrapolations as seen in Figure 3b,c. The 95% confidence intervals of the forecasted number of new cases in these figures gives clear indication that one could rely on such methodology with a high level of certainty for at least the first 1–2 months of the extrapolation, a critical period for control of the outbreak. It is very possible to improve the performance of our forecast by using weights to account for the number of conducted daily tests. However, we intended to rely on a flexible mathematical modeling scheme with four parameters to give a simple approximation for two extremes while noting that better predictions are expected as the pandemic unfolds and more data become available.

## 5. Conclusions

The present COVID-19 pandemic is a new and rapidly evolving outbreak which has been responded to and contained to varying degrees by countries around the world. While a number of non-modifiable factors likely explain the success, or lack thereof, of jurisdictional mitigation and containment efforts, it is highly likely that differences in public health actions also play a significant role, allowing communities anticipating the virus to learn and adapt from action plans that have proven effective. In the current study, we identify a three-month variation in the likely length of the pandemic, a 1.5-fold difference in the number of daily infections, and a 3-fold difference in the expected cumulative cases when applying the experience of two developed countries in handling this virus to the Utah context. These variations, and the public health, morbidity, and mortality impacts they connote, both describe the possible distribution of outcomes for one U.S. state, as well as provide a cautionary warning about which public health actions must be urgently emulated and adapted to minimize the impact of the epidemic, and which are likely to result in local catastrophe.

## Figures and Tables

**Table 1:** New cases of COVID-19 in Utah, Italy, and South Korea by date with daily rates of spread.

Date	Utah	Italy		South Korea	
	New Cases	New Cases	Daily Spreadrate	New Cases	Daily Spreadrate
2/15/2020	0	0		0	
2/16/2020	0	0		1	
2/17/2020	0	0		1	
2/18/2020	0	0		1	
2/19/2020	0	0		27	
2/20/2020	0	1		53	
2/21/2020	0	17		98	
2/22/2020	0	58		227	
2/23/2020	0	78		166	
2/24/2020	0	72		231	
2/25/2020	0	94		144	
2/26/2020	0	147		284	
2/27/2020	0	185		505	
2/28/2020	1	234		571	
2/29/2020	0	239		813	
3/1/2020	0	573		586	
3/2/2020	0	335		599	0.0222
3/3/2020	0	466		851	0.4207
3/4/2020	1	587		435	−0.4888
3/5/2020	1	769		663	0.5241
3/6/2020	3	778	0.0117	309	−0.5339
3/7/2020	2	1247	0.6028	448	0.4498
3/8/2020	1	1492	0.1965	272	−0.3929
3/9/2020	0	1797	0.2044	165	−0.3934
3/10/2020	1	977	−0.4563	35	−0.7879
3/11/2020	3	2313	1.3675	242	5.9143
3/12/2020	5	2651	0.1461	114	−0.5289
3/13/2020	6	2547	−0.0392	110	−0.0351
3/14/2020	14	3497	0.3730	107	−0.0273
3/15/2020	11	3590	0.0266	76	−0.2897
3/16/2020	8	3233	−0.0994	74	−0.0263
3/17/2020	25	3526	0.0906	84	0.1351
3/18/2020	13	4207	0.1931	93	0.1071
3/19/2020	35	5322	0.2650	152	0.6344
3/20/2020	31	5986	0.1248	87	−0.4276
3/21/2020	38	6557	0.0954	147	0.6897
3/22/2020	78	5560	−0.1521	98	−0.3333
3/23/2020	32	4789	−0.1387	64	−0.3469
3/24/2020	37	5249	0.0961	76	0.1875
3/25/2020	68	5210	−0.0074	100	0.158
3/26/2020	97	6203	0.1906	106	0.0600
3/27/2020	116	5909	−0.0474	91	−0.1415
3/28/2020	114	5974	0.0110	146	06.044
3/29/2020	83	5217	−0.1267	105	−0.2808
3/30/2020	119	4050	−0.2237	78	−0.2571
3/31/2020	92	4053	0.0007	125	0.6026
4/1/2020	99	4782	0.1799	101	−0.1920
4/2/2020	169	4668	−0.0238	89	−0.1188
4/3/2020	128	4585	−0.0178	86	−0.0337
4/4/2020	137	4805	0.0480	94	0.0930

**Table 2:** Timeline of epidemic development and public health actions in South Korea and Italy.

Date	South Korea	Italy	Utah
22-January	First reported case of Coronavirus [16]		
31-January		First reported cases of Coronavirus [16]	
3-February	Korean-made diagnostic test approved for use [17]		
20-February		Cluster in Lombardy Region identified [18]	
21-February	Public spaces closed and protests banned [19]		
23-February	Govt puts nation on highest alert, “Grave”, allowing order of temp closure of schools, reduction of travel, including public transport and flights to/from S. Korea [20]	11 municipalities in Lombardy quarantined [18]	
26-February		Announces relaxation of testing: connections to confirmed cases or recent travel to affected areas, testing upon onset of symptoms [15]	
29-February	Drive-through testing widely available [21]		
3-March		10% of medical workers in Lombardy diagnosed w/COVID-19 [22]	Univ. of Utah & Southern Utah Univ suspend study abroad [23]
4-March		Order closure of all schools and universities [22]	
6-March			First reported case of Coronavirus; Governor declares State of Emergency [24,25]
8-March		Gov’t restricts movement to and from Lombardy region [18]	
9-March		Nation placed under quarantine orders travel ban [26]	
11-March		Order closure of businesses excl grocery and pharmacies [27]	Governor calls for 100-person limit at public gatherings [28]
13-March		20% of medical workers in Lombardy are diagnosed w/COVID-19; some have died [18]	
14-March	274,504 tests for Coronavirus completed [29]		First documented case of community spread; Governor announces 2-week soft closure of public schools [30]
16-March	Begin screening every person arriving at airports, incl citizens [31]		Summit and Salt Lake County announce closure of most public gathering places [32]
17-March	Gov’t offers small subsidy (~$360) per month to anyone self-isolating, regardless of pos/neg test [31]		Governor halts in-restaurant dining; Utah officials tell patients not to seek testing [33]
18-March			Lt. Governor makes public statement that no state-wide isolation orders are being considered [34]
19-March	Reports 307,000+ tests conducted [35]	Becomes nation with highest COVID-19-related deaths in the world [36]	Large-scale testing begins [37]
20-March		475 fatalities; gov’t outdoor exercise; military dispatched to Lombardy to ensure compliance with lock-down measures (9888 infractions for non-compliance) [36]	
21-March	3.8 trillion Korean won committed for disaster mgmt. funding, focusing on small business and disadvantaged people, particularly those with COVID-19-related difficulties [31]	2857 people in ICU, up from 2655 on 3/20 [36]	
23-March			School closures extended until May 1 [38]
25-March			Summit County and Navajo Nation issue stay-at-home orders [39,40]; community spread confirmed in Washington County [41]
27-March			Governor issues directive asking for voluntary self-isolation [42]; Salt Lake City Mayor issues stay-at-home directive [43]
1-April			Governor announces suspension of evictions for Coronavirus-related non-payment of rent until May 15 [44]
